# Supplementary material for: Iterative improvement in the automatic modular design of robot swarms
Source: PeerJ Comput Sci. 2020 Dec 7;6:e322. doi: 10.7717/peerj-cs.322 (PMC7924708; doi:10.7717/peerj-cs.322)
Supplement: Supplemental Information 3 [file peerj-cs-06-322-s003.zip › argos3/doc/api/standalone/a00657_source.html]

ARGoS: plugins/simulator/visualizations/qt-opengl/qtopengl\_render.h Source File


- Main Page
- Related Pages
- Namespaces
- Classes
- Files

- File List
- File Members

# plugins/simulator/visualizations/qt-opengl/qtopengl\_render.h

Go to the documentation of this file.

```
00001 
00007 #ifndef QT_OPENGL_RENDER_H
00008 #define QT_OPENGL_RENDER_H
00009 
00010 namespace argos {
00011    class CQTOpenGLRender;
00012    class CQTOpenGLApplication;
00013 }
00014 
00015 #include <argos3/core/simulator/visualization/visualization.h>
00016 #include <argos3/plugins/simulator/visualizations/qt-opengl/qtopengl_main_window.h>
00017 
00018 #ifdef ARGOS_WITH_LUA
00019 #include <argos3/plugins/simulator/visualizations/qt-opengl/qtopengl_lua_main_window.h>
00020 #endif
00021 
00022 namespace argos {
00023 
00024    class CQTOpenGLRender : public CVisualization {
00025 
00026    public:
00027 
00028       CQTOpenGLRender() :
00029          m_pcApplication(NULL),
00030          m_pcMainWindow(NULL),
00031          m_ppcOptions(NULL),
00032          m_nOptionNum(0)
00033 #ifdef ARGOS_WITH_LUA
00034          , m_pcQTOpenGLLuaMainWindow(NULL),
00035          m_bLuaEditor(false)
00036 #endif
00037       {}
00038 
00039       virtual ~CQTOpenGLRender() {}
00040 
00041       virtual void Init(TConfigurationNode& t_tree);
00042 
00043       virtual void Execute();
00044 
00045       virtual void Reset() {}
00046 
00047       virtual void Destroy();
00048 
00049       CQTOpenGLMainWindow& GetMainWindow();
00050 
00051 #ifdef ARGOS_WITH_LUA
00052       CQTOpenGLLuaMainWindow& GetLuaMainWindow();
00053 #endif
00054 
00055    private:
00056 
00057       CQTOpenGLApplication* m_pcApplication;
00058       CQTOpenGLMainWindow* m_pcMainWindow;
00059       char** m_ppcOptions;
00060       SInt32 m_nOptionNum;
00061       TConfigurationNode m_tConfTree;
00062 #ifdef ARGOS_WITH_LUA
00063       CQTOpenGLLuaMainWindow* m_pcQTOpenGLLuaMainWindow;
00064       bool m_bLuaEditor;
00065 #endif
00066 
00067    };
00068 
00069 }
00070 
00071 #endif
```

---

Generated on 10 Jul 2018 for ARGoS by 
 1.6.1
